# Barriers and facilitators to ART adherence among ART non-adherence people living with HIV in Cameroon: A qualitative phenomenological study

**DOI:** 10.1371/journal.pone.0291487

**Published:** 2023-09-12

**Authors:** Amos Buh, Raywat Deonandan, James Gomes, Alison Krentel, Olanrewaju Oladimeji, Sanni Yaya

**Affiliations:** 1 Interdisciplinary School of Health Sciences, University of Ottawa, Ottawa, Ontario, Canada; 2 School of Epidemiology and Public Health, University of Ottawa, Ottawa, Ontario, Canada; 3 Department of Public Health, Faculty of Health Sciences, Walter Sisulu University, Mthatha, Eastern Cape, South Africa; 4 Faculty of Health Sciences, Durban University of Technology, Durban, South Africa; 5 School of International Development and Global Studies, University of Ottawa, Ottawa, Ontario, Canada; 6 The George Institute for Global Health, Imperial College London, London, United Kingdom; University of KwaZulu-Natal College of Health Sciences, SOUTH AFRICA

## Abstract

**Background:**

Antiretroviral therapy (ART) needs to be taken for life with near perfect levels of adherence for it to be effective. Nonetheless, ART non-adherence is still observed in sub-Saharan African (SSA) countries such as Cameroon. The objective of this study was to assess the factors influencing non-adherence and or adherence among people living with HIV (PLWH) who have experienced non-adherence to ART in Cameroon.

**Methods:**

A descriptive qualitative study of PLWH who have experienced non-adherence with ART in Cameroon was conducted. Data were collected using in-depth interviews. Collected data were analyzed using the NVIVO 12 software.

**Results:**

In total, 43 participants participated in this study. The Southwest and Littoral regions each contributed 15 (34.88%) of participants, participants’ mean age was 37.1 years (SD: 9.81) and majority 34 (82.93%) were females. ART adherence barriers include those related to patient (forgetfulness, business with other things, unwillingness to swallow drugs daily), medication (side effects), health service (arrogance of caregivers, occasional drug shortages at treatment centre, poor counseling of patient), stigma (fear of status disclosure), use of alternative treatment (traditional medicine, prayers and deliverance), resource limitation (limited food, limited finances), environmental/social (limited or no home support), and political instability (disruption of free circulation by ghost towns, roadblocks and gunshots in some regions). ART adherence facilitators include social support (family and peer support), aligning treatment with patient’s daily routines (align ART with schedule of family members), use of reminders (phone alarm, sound of church bell), health sector/caregiver support (messages to patient, financial support, proper counseling), and patient’s awareness of HIV status/ART knowledge (awareness of HIV positive status, Knowledge of ART benefits).

**Conclusion:**

ART adherence barriers in Cameroon include those related to patient, medication, health service, stigma, use of alternative treatment, resource limitation, environmental/social, and political instability. ART adherence facilitators include social support, aligning treatment with patient’s daily routines, use of reminders, health sector/caregiver support, and patient’s awareness of HIV status/ART knowledge. Given these barriers and facilitators, continuous information provision and consistent support both from patients’ families and caregivers are needed to improve adherence among patients. Further studies including many regions and larger samples using both in-depth and focused group discussions as well as quantitative approaches are required to uncover the burden related to ART non-adherence.

## Introduction

Antiretroviral therapy (ART) is typically a combination of three or more drugs that interrupts the Human Immunodeficiency Virus (HIV) replication, enables immune recovery and improves survival among people living with HIV (PLWH) [1]. However, poor retention in care and non-adherence to ART continues to undermine the success of HIV treatment and care programs across the world, especially in sub-Saharan Africa (SSA) where the burden of HIV is heaviest [2–4].

When a person is infected with HIV, the virus targets the immune system and weakens the individual’s defense against other infections. As the virus progressively destroys and impairs the functions of the immune cells, infected individuals become immunodeficient and their susceptibility to many infections, cancers and other diseases increases [5]. To avoid a progressive destruction of the immune cells, it is important that people should be effectively linked to care once they are diagnosed as HIV-positive [6].

At the moment, there is no cure discovered for HIV; the only treatment modality to prolong life and to improve quality of life of PLWH is ART [7, 8]. ART has revolutionized the course of the HIV disease, transforming the HIV infection from a life-threatening infection to a manageable chronic condition [4]. It prevents further replication or multiplication of the virus, reduces the patient’s viral load, increases CD4 counts, reduces the patient’s likelihood of opportunistic infections and hospitalizations, improves patient’s quality of life and reduces HIV related morbidity and mortality [9–11]. The increase in the patient’s CD4 cell count allows the individual’s immune system to recover and produce more CD4 cells which fights off infections and other HIV-related cancers [7]. To be effective however, ART has to be taken life-long as prescribed, with near perfect levels of adherence [8, 12]. Adherence to ART reduces the viral load in an individual’s body, prevents treatment failure and the likelihood of the emergence of drug-resistant strains of the virus and also prevents further transmission of the virus to non-infected persons [1, 7, 13, 14]. In fact, with timely access to ART, PLWH can expect a near normal life expectancy [1, 13, 14]. Despite this, most health care systems in SSA face numerous challenges emanating from scaling-up ART for PLWH–prominent among these challenges are poor retention in care, congestion of the primary health care facilities and sub-optimal adherence to ART [3].

The barriers to ART adherence have been established in some studies. The identified major obstacles to ART adherence include stigma, negative perception, and lack of family and community support [15, 16]. Other factors such as disclosure of status, unemployment, lack of transport to go to the health facility for ART, insufficient feeding, inadequate follow ups, lack of patient confidentiality, lack of disability grants and alternative forms of therapy have also been reported as common barriers to ART adherence [16]. Besides these, physical, economic and emotional stresses, travel from home, business outside the home, depression, alcohol or drug use and ART dosing frequency have also been reported as barriers to adherence [17, 18].

Some studies have also reported facilitators to ART adherence including social support [19, 20], responsibility for raising children, disclosure of HIV status, improved health on ART, use of reminder aids and receiving education and counseling [19, 21]. Community knowledge and understanding of the HIV infection, increasing collaboration between Western and Traditional providers, peer and family level support, decreasing cost and distance to ART clinic [22] as well as clear instructions for taking ART, service providers’ positive attitude towards patients, benefits of adhering to ART and dangers of defaulting [21] have also been documented as factors facilitating ART adherence. Nevertheless, evidence is limited about the specific barriers and facilitators to ART adherence that are common in some SSA and or low- and middle-income countries such as Cameroon. More specifically, it is hard to find literature on the views of patients who have actually experienced non-adherence with HIV treatment concerning their barriers and facilitators to treatment adherence. As such, information for policy development targeting specific ART adherence barriers in order to improve HIV control and management is limited.

Also, Cameroon has been undergoing a political crisis since 2016. It has been documented that political conflicts leads to disruption of health care and displacement of patients [23]. Besides political conflicts, the COVID-19 pandemic has also been reported to have disrupted HIV care [24]. Thus, the political instability in Cameroon and the COVID-19 pandemic might have further limited access to ART for PLWH in this country. The objective of this study therefore was to assess the factors that influence non-adherence and or adherence to HIV treatment in PLWH who have experienced HIV treatment non-adherence in Cameroon.

## Methods

### Study design

A qualitative phenomenological study design was used in this study. This design was conducted on adult PLWH who have experienced ART non-adherence in Cameroon.

### Research setting

Briefly, the study was conducted in Cameroon–a country in sub-Saharan Africa with a high prevalence of HIV. The country has a population of over 28 million inhabitants [25] and it is divided into ten regions. Participants for the study were enrolled in HIV treatment centres in three selected regions (Littoral, Southwest and Northwest regions–all selected through balloting; this process involved making a list of the ten regions of the country, writing them on pieces of paper in a basket and then randomly picking three regions for the study) of the country. In each of the selected regions, the study was conducted in only one HIV treatment centre that was purposefully selected based on probability proportionate to the size of patients registered in the centre.

### Study population, participants and sampling

This study only included PLWH who were on ART and had experienced ART non-adherence in Cameroon. To be eligible for the study, a participant had to be a PLWH receiving treatment in a treatment centre in the country, be at least 21 years old, have been on treatment for at least six months and have given their consent to participate. Furthermore, this study only included PLWH who had experienced HIV treatment non-adherence in Cameroon. PLWH who were severely sick, having poor mental health, or had other ailments or disabilities that prevented them from providing the study with the requested information were excluded from the study.

We anticipated recruiting up to 30 participants in each centre. However, the number of participants in this study was largely dependent on responses received from participants. As long as there was variation in participant opinions, more participants were recruited. Recruitment of new participants in each centre (after recruiting a minimum of 10 participants per centre) stopped once saturation was attained. Overall, a total of 43 participants were enrolled in the study between the period of November and December 2021.

The sampling and enrollment of participants in this study was done by six trained interviewers (two from each of the selected regions where the study was conducted) with a background training in health. The purposive sampling technique was employed by study interviewers to recruit participants in each study centre. Only PLWH who had experienced non-adherence (missed refill, ART appointment, taking ART as prescribed and had detectable viral loads) with ART in any of the three selected study centres were purposefully retained to participate in the study. This procedure continued until saturation was reached in each centre.

### Data collection

To collect data for this study, in-depth interviews (IDIs) were conducted on PLWH who had experienced non-adherence with ART. Open ended questions adapted from similar studies [26, 27], were used to collect data on participants’ perceptions about factors contributing to their non-adherence to ART. The interviews were conducted in French or English by trained interviewers. To ensure privacy, all interviews were conducted in an arranged office in each treatment centre and participants’ responses to questions were recorded.

Prior to analysis, the audio-recorded data were transcribed word verbatim and coded into common themes with the assistance of translators. This was cross-checked by other data collectors and one of the investigators for consistency, to ensure that strict methodological rigor was followed.

### Data analysis

The NVIVO 12 software was used to analyze data. The collected data were transcribed and coded into themes and sub-themes for thematic analysis, as per Braun & Clarke’s [28] guide for conducting a thematic analysis. This guide was used because it allowed for a transparent and rigorous analysis that produced relevant information for this study. Two people independently read the transcripts multiple times to immerse themselves in the raw data and made notes on initial topics and ideas relevant to the research question. The transcripts were coded iteratively, returning to the transcripts and altering and modifying the codes in response to the data and emerging patterns. Because coding word-by-word or line-by-line limits the ability to see patterns among and between pieces of data [29], lines of text from transcripts were broadly coded, ranging from a sentence to several sentences, so that participants’ intentions were not lost. Finally, the two independent coders discussed their codes and reached a consensus agreement to resolve any differences. The codes were then used to create themes and subthemes that would respond to study objectives. The sub-themes were organized into major themes, and then each theme was described.

### Trustworthiness

In conducting a qualitative study, trustworthiness is crucial to ensure that the study is rigorous and that it produces findings capable of impacting policy and practice [30]. To ensure trustworthiness in this study, trained personnel were recruited to collect data, transcribe, code and analyze the data. One of the investigators (AB) closely monitored the data collection process to ensure that data were properly collected. Following data collection, two independent individuals (AB and IKK) transcribed and coded the data and then, they discussed their codes together in order to reconcile any differences. Two investigators (AB and SY) then reviewed the coded data in order to validate it and ensure that the codes and themes resulting from the transcripts were credible. Any issues such as contradictions, thematic misinterpretations and factual errors discovered by the investigators were discussed and clarifications made. An external auditor (CBM) skillful in qualitative research together with one of the investigators (SY) reviewed the coded data to further ensure dependability.

Also, to ensure that findings reflected the reality of the barriers to ART adherence, data were only collected from people who had actually experienced ART non-adherence. The data were collected across three regions of the country and this range of data helped enriched and deepened understanding of study findings.

#### Ethical considerations

Ethical clearance for the study was obtained from the Health Sciences and Science Research Ethics Board (REB) of the University of Ottawa (Ethics File Number: H-08-21-7274) and the Cameroon Baptist Convention Health Board Institutional Review Board (CBCHBIRB) (IRB study number: IRB2021-53). Administrative authorization was also obtained from the Regional Delegate of Public Health of the Southwest Region (Ref: 1211/MINSANTE/SWR/RDPH/P5/810/725) and the Regional Hospital Buea (Ref /MPH/SWRDPH/BRH/IRB). Written informed consent was obtained from all participants prior to their participation in the study.

### Inclusivity in global research

Additional information regarding the ethical, cultural, and scientific considerations specific to inclusivity in global research is included in the S1 Checklist.

## Results

### Participants’ characteristics

**Fig 1:**
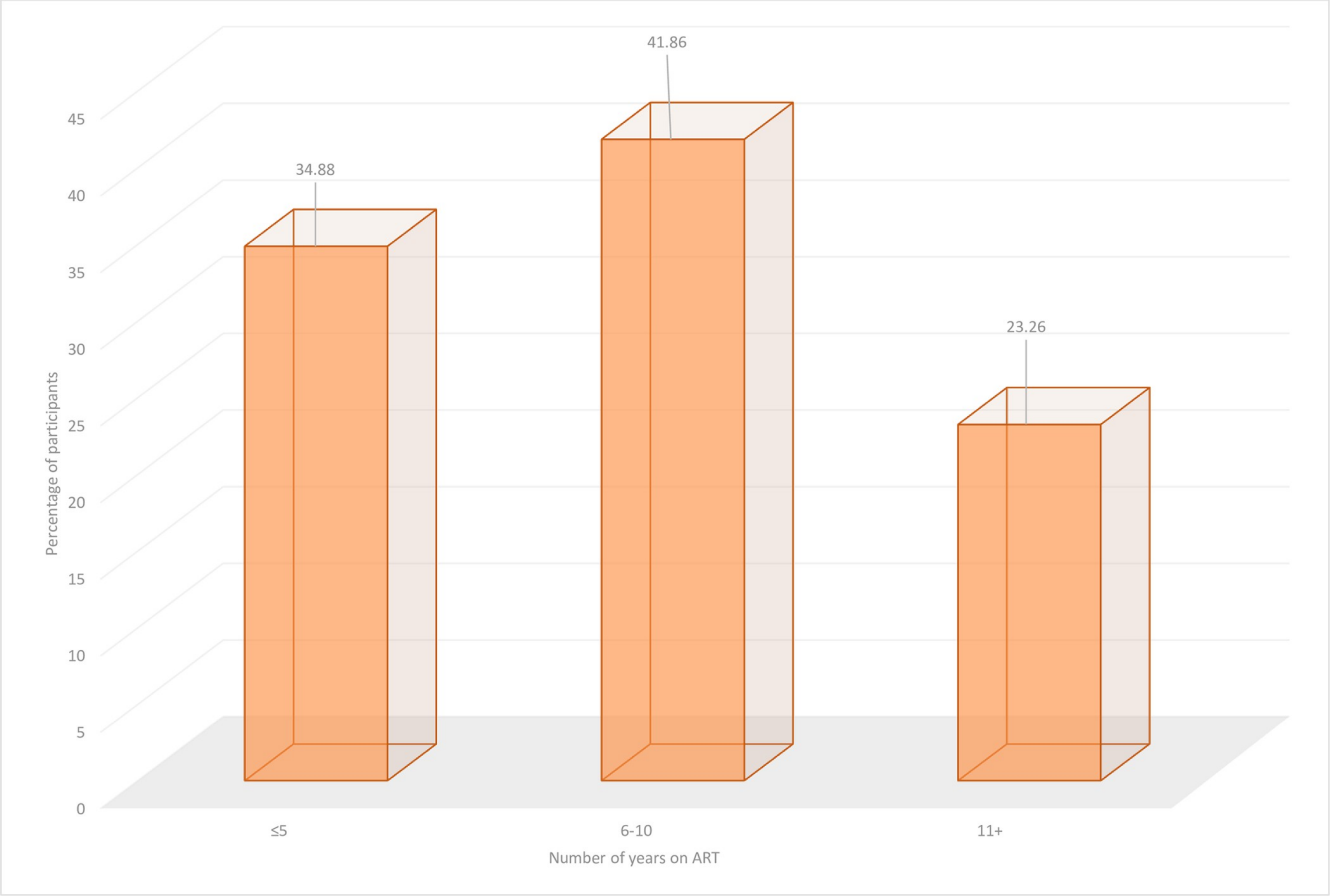
Proportion of participants versus length of time on ART.

The characteristics of the 43 participants that took part in this study are presented in Table 1. The Southwest and Littoral regions of the country each contributed 15 participants in the study and the mean age of participants was 37.1 years (SD: 9.81). Most participants (34) in the study were females. Single and married participants each represented more than a third (16) of participants in the study. More than half (24) of the participants attained only the secondary level of education and less than one third (14) of them were Roman catholic Christians. Few (11) of the participants were engaged in business activities and the ART dosing frequency for all participants in the study was once daily. The median number of years participants had been on ART was 7 years (IQR: 3–10); in fact, more than one third (41.86%) of participants had been on ART for 6–10 years (Fig 1).

**Table 1:** Characteristics of the overall study population (N (%) or mean (SD) or median (IQR)).

Characteristic	N or Mean or Median	% or SD or IQR
Region of residence		
Northwest	13	30.23
Southwest	15	34.88
Littoral	15	34.88
Age	37.1	9.81
Sex		
Male	7	17.07
Female	34	82.93
Marital status		
Single	16	38.10
Married	16	38.10
Divorced	1	2.38
Separated	3	7.14
Widowed/widower	6	14.29
Education*		
Primary	13	30.95
Secondary	24	57.14
High school	1	2.38
University	4	9.52
Religion		
Muslim	2	4.76
Catholic	14	33.33
Baptist	4	9.52
Presbyterian	7	16.67
Pentecostal	13	30.95
Animist	2	4.76
Occupation		
Business	11	27.50
Cleaner	1	2.50
Designing	1	2.50
Driving	2	5.00
Entrepreneur	1	2.50
Farming	9	22.50
Hairdressing	1	2.50
Housewife	4	10.00
Mechanic	1	2.50
Nursing	1	2.50
Retired	1	2.50
Security officer	1	2.50
Student	2	5.00
Tailoring	2	5.00
Teaching	2	5.00
ART years	7	3–10
ART daily dosing		
Once daily	43	100.00

*Primary education involves at most seven years, secondary at most twelve, high school at most fourteen and university above fourteen years of education. N = frequency, % = frequency in percentage, SD = standard deviation and IQR = inter quartile range.

### Barriers to ART adherence among ART non-adherent participants in Cameroon

Table 2 shows the details of the barriers to ART adherence among ART non-adherent participants. The barriers are separated into main themes and are described with some participants’ excepts in quotations as follows:

**Patient related barriers:** These are patients’ behavioral factors or activities that prevents them from taking their medication. They are linked to the fact that patients displace themselves without their drugs either to the farm or other occasions and spend a night there or return too late for them to take their drugs. Added to this, some patients feel tired of taking drugs daily and others simply forget to take them.

“*Nothing hinders me except that I forget because if I forget I know that I have passed that day without taking it so it is only for the next day that I will take it*.*” [56 years old*, *female]*.

“*I don’t like swallowing medicines every day*, *each time I take it*, *I ask God*, *when is this going to end*.*” [36 years old*, *female]*.

“*I went for a burial and slept there and came back to the house when 6A*.*M had already passed so I could not take it again*.*” [36 years old*, *female]*.

**Medication barriers:** These are drug reactions on the patients when they swallow the drugs. These are mostly due to side effects of the Antiretroviral drugs (ARVs) such as nausea, dizziness and nightmares experienced by some patients when taking the drugs.

“*I think the most difficult part is that when I go to work the medicine makes me weak*.*” [40 years old*, *male]*.

“*Well*, *when I started taking the medicine it used to turn my neck*, *it used to make me feel like I will vomit as if I was pregnant*, *but it was just when I started the medicine” [66 years old*, *female]*.

“*I used to have so many dreams at night especially about death when I started taking the medicines*, *but after a while this stopped*.*” [38 years old*, *female]*.

**Health service barriers:** These are barriers encountered by patients in the healthcare facilities which prevents or discourages patients from taking their ART. These stem from drug shortages to caregiver attitudes which cause patients to wait for long periods of time when they go to collect their drugs in treatment centres. This makes some patients feel frustrated and they will abandon their ARVs. Besides this, poor counseling of patients on importance of adhering to their treatment makes patients non-adherent to ARVs.

“*Some of the nurses are harsh*, *like when somebody just comes in*, *they’ll be like “come*, *na ya book this*? *(Come*, *is this your book*?*)*.*” [26 years old*, *female]*.

“*The hardest part is when I come to the hospital like this to come and take it*, *and when they are delaying to give my medicine like that*, *it really bothers me*.*” [33 years old*, *female]*.

**Stigma related barriers**: Fear of disclosure of status makes some patients feel ashamed of moving around with their drugs or even taking them in the midst of others. As such, they leave drugs at home when they travel and even when they are in possession of the drugs, they would not take them if they were in the company of others.

“*I feel uneasy to take it because when I travel like that*, *maybe I may go with people I feel uneasy taking the drugs in the midst of people*.*” [35 years old*, *female]*.

“*Number 1 problem is forgetfulness and number 2 is that whenever I’m elsewhere and it is time for drugs*, *I cannot remove it to drink when people are around*.*” [45 years old*, *male]*.

**Alternative treatment**: The belief that traditional medicine cures a patient of HIV or that prayers to God could make a patient receive deliverance from HIV renders some patients non-adherent to their ARVs when they go in search for this alternative treatment.

“*I stopped taking it for some time because I believed that God has the final say*, *God is the healer*, *and he can do anything to be possible so that made me to stop taking it for some time*.*” [47 years old*, *male]*.

“*I don’t have anything that is really difficult for me*, *it’s just that I’m not really happy that every day I have to take them because it is showing that what we are taking are chemicals which aren’t good for humans … at times I feel like I should even go for these herbs*, *I have a specialist I went to him to try to treat it and he showed me some amount of people that have come to him concerning HIV*, *it’s just that it is expensive*.*” [38 years old*, *female]*.

**Resource limitation**: Patients are often advised to eat well before swallowing their ARVs; when there is neither food nor money to buy food, some patients simply abandon their drugs. Besides this, some patients need money to pay transport to the treatment centre to collect/refill their drugs; in case of lack of money, they simply stay home and cannot continue taking their drugs when the drugs get finish.

“*The most difficulty I face when taking my medicine is when I don’t have the transport to go and take it every day*. *That is the only difficulty*.*” [34 years old*, *male]*.

“*As I am taking this medicine*, *they said I should be eating fruits*, *vegetables*, *and many other things but with this crisis someone is just short of money*, *you see food*, *but you are not able to buy it*.*” [47 years old*, *female]*.

**Environmental/social barriers**: Some patients are faced with multiple problems–intermittent lack of support for child care as well as limited transportation means to go to centre for treatment. For instance, some of the patients’ spouses or family members who provide support to help them take their drugs occasionally travel–leaving the patients with no support and at such moments, patients are non-adherent to their drugs.

“*At times I forget my rendezvous like when my husband travels*, *I have difficulty in coming because it is far plus with the children so at times my rendezvous comes*, *and he is not there*, *and he’s the one who always carries me to come here*, *so*, *when he travels like that*, *to come here it is difficult with the kids; that is why at times it disturbs me*.*” [21 years old*, *female]*.

**Poor mental health:** Some patients are suffering from poor mental related health issues such as depression. Such patients find no meaning/reason in living or being alive. This makes them lose hope on their treatment to the point where they give up on taking ART in preference to death.

“*See it’s because I lost my children and I also lost my workshop*, *that’s where I just gave up*, *it’s not like I was ignorant*. *I just wanted to give up that it is just better for me to die*.*” [33 years old*, *male]*.

**Intrapersonal and cognitive barriers:** Some patients have a hard time seeing themselves as the only one taking ART in the house or family. These patients even when they have social support or people to help remind them adhere to their treatment, they perceive the help as unwanted because those providing the help are not taking the treatment.

“*The most difficult part is that everyone in the house is not taking it and you’re the only one taking it; so*, *like when*, *might be someone will remind you*, *like you just get angry that this person is not taking it and the person is telling me to go and take my medicine*, *it doesn’t concern the person like don’t remind me to take them*.*” [21 years old*, *male]*.

**Political instability**: Two of the regions (Northwest and Southwest regions) where the study was conducted are experiencing an ongoing political crisis–there are sometimes roadblocks, gunshots, and organization of ghost towns (days in which all activities including movement of people in a city are closed down) on some days which obstructs circulation and scares patients from going to collect/refill their drugs when they are finished. When patients run out of drugs during these periods, they have no option but to stop taking treatment.

“*The challenge I have is only that the last time I went to the village*, *I overstayed there which wasn’t my plan because they blocked the roads*, *so I wasn’t able to come back*, *that is the only challenge I have*.*” [31 years old*, *male]*.

“*… because of these gunshots we can run to a different quarter to go and sleep*, *so the thing disturbed me and just that one day that I didn’t take my medicine it disturbed me a lot*.*” [47 years old*, *female]*.

“…with all these ghost towns at times we make 2weeks at home without going anywhere and my medicine got finished.” *[37 years old*, *female]*.

“*… I went to make 2weeks in the village and they locked the roads*, *so I made one month and did not take medicine for 2 weeks*.*” [42 years old*, *female]*.

**Table 2:** Barriers to ART adherence among people who have experienced ART non-adherence in Cameroon.

Themes	Sub-themes	Example
Patient	• Forgetfulness • Business with other things • Hate swallowing drugs	• Went to the farm or work or occasion and unexpectedly slept there or returned late • Feeling tired or not comfortable swallowing drugs daily
Medication	• Side effects	• Nausea • Dizziness • Nightmares
Health service	• Arrogant caregivers • Drug shortages • Poor counseling	• Delay in dispensing drugs to patient when they are in hospital • Unwelcoming attitudes of some caregivers
Stigma related	• Fear of status disclosure	• Carrying drugs around or taking them in public can make people discover patient’s status and stigmatize them
Alternative treatment	• Traditional medicine • Prayers and deliverance	• Belief that traditional medicines flushes out virus from one’s system • Belief that God can deliver one from illness if they focus on prayers and fasting
Resource limitation	• Limited food • Limited or no finances	• Did not have food to eat before taking drug • Lacked money for transportation to hospital
Environmental/social	• Limited or no home support	• Live alone/spouse travels
Poor mental health	• Depression	• See no meaning in being alive
Intrapersonal and cognitive	• Lone person on ART	• See self as the only one taking ART in family
Political instability	• Disruption of movement by ghost towns	• Roadblocks, gunshots on ghost town days scares people to go for drugs

### Facilitators to ART adherence among ART non-adherent participants in Cameroon

Table 3 shows factors helping patients’ adherence to ART. Major factors facilitating ART adherence among patients include:

**Social support**: Most patients would adhere to treatment if they were supported emotionally, materially, and financially by their family members and or peers.

“*At times when I forget my rendezvous*, *it is my husband who reminds me himself*, *he even brings me here to collect my medicines when I have my rendezvous*. *He is really helping me take my medicines*.*” [32 years old*, *female]*.

**Patients’ daily routines**: Aligning the time patients are supposed to take their drugs to coincide with the time certain routine schedules occur helps patients remember to take their drugs. For instance, taking drugs before spouse/family member leaves for work every morning or after brushing teeth in the morning or just when children are preparing to go to school.

“*When I wake up and brush*, *I just take my medicine and I eat well*, *if there is fruit to eat I will eat on top of it*.*” [34 years old*, *female]*.

**Reminders**: Setting up an alarm on a phone at a particular time when drugs should be taken or programing to take drugs when the church bell rings, for patients staying besides the church or when children are about going to school helps some patients remember to take their drugs.

“*I’m just beside the church so when they ring the bell I just wake up and take it*.*” [38 years old*, *female]*.

“*… Once I hear the alarm on my phone ring every morning it just indicates that I have to take it*.*” [30 years old*, *female]*.

**Health sector/caregiver support**: Some patients expressed that, messages, or calls from caregivers reminding them to go for their drugs helps them adhere to treatment. Others state that availability of drugs at the treatment centre at all times, development of alternative ART drug formulations (such as injectables) and provision of financial incentives to assist in patient transportation to go collect drugs at the treatment centre, would help in treatment adherence.

“*The only way you people can help me is that if you have a chance*, *you can call me at that time to remind me so that if I was elsewhere*, *I could say eh my nurse just biped me that I should take my medicine because there are times one sleeps and forget*.*” [47 years old*, *male]*.

“*It’s just the finances you people could help me with because for the moment I’m not doing anything so coming to take drugs is not easy for me*.*” [32 years old*, *female]*.

“*Well*, *what could help ease the task for me is if the drugs could be given to me through injection*.*” [40 years old*, *female]*.

**Patient’s awareness of health status/ART knowledge**: Some participants reported that being aware of their HIV status and knowing that ART improves their health, motivates them to continue taking the drugs as prescribed.

“*I know that the medicine is a tablet that’s like it’s not that it cures the virus that is in us*, *but it makes the virus to be weak in a way that it cannot multiply because as long as I am not taking my medicine*, *the virus multiplies every day plenty*. *So*, *when I am taking the medicine*, *it makes the virus to lose that energy or force to multiply*, *so it makes it weak*, *and it just stays in one place*. *I know that I have the virus*, *but it is that the virus cannot multiply as long as I drink my medicine*.*” [27 years old*, *female]*.

**Table 3:** Factors that facilitate ART adherence among people who have experienced ART non-adherence in Cameroon.

Themes	Sub-themes	Example
Social support	• Family support • Peer support	• Provide emotional and material/financial support • Help reminding patient to take drugs
Patients’ daily routines	• Align ART with recurring routines • Align ART with schedule of family members	• Patient takes drugs when children are preparing to go to school or before spouse/family member goes to work
Reminders	• Phone alarms • Church bell • Living with pupils/students	• Patient is reminded of time to take drugs when phone alarms or when church bell rings or when children wakeup to go to study
Health sector/caregiver support	• Messages/calls to patient • Financial support • Proper counseling • Availability of drugs • Alternative drug formulation (injectables)	• Text messages or calls reminding patients to go for their drugs • Continuous education of patients on importance of adhering to treatment
Patient’s awareness of health status/ ART knowledge	Aware of HIV status • Know ART helps	• experience of an improvement in health with ART

## Discussion

Non-adherence or sub-optimal adherence to ART may result in unsuppressed viral load, leading to development of drug resistance, opportunistic infections and ultimately mortality of patient [27, 31]. To ensure prolonged benefits of treatment therefore, adherence to ARVs is key [12, 27, 32].

We assessed the barriers to ART adherence among people who have experienced ART non-adherence, as well as the factors that facilitate ART adherence among these people, in three regions of Cameroon (Northwest, Southwest and Littoral regions). We have documented that the barriers to ART adherence in Cameroon are classified into ten major themes: patient barriers (forgetfulness, other commitments, unwillingness to swallow drugs on a daily basis), medication barriers (drug side effects), health service barriers (arrogance of caregivers, occasional drug shortages at treatment centre, poor patient counseling), stigma-related barriers (fear of status disclosure), use of alternative treatment (traditional medicine, prayers and deliverance), resource limitation (limited food, limited finances), environmental/social barriers (limited or no home support), poor mental health barriers (depression), intrapersonal and cognitive barriers (seeing self as the only one taking ART) and political instability (disruption of free circulation by ghost towns, roadblocks and gunshots in some regions). We identified five major groups of ART adherence facilitators: social support (family and peer support), aligning treatment with patient’s daily routines (align ART with family members’ schedules), use of reminders (phone alarm, sound of church bell), health sector/caregiver support (messages to patient, financial support, proper counseling), and patient’s awareness of HIV status/ART knowledge (awareness of HIV positive status, knowledge of ART benefits).

While the barriers to ART adherence in this study represent the reality of study participants living in Cameroon, they reflect ART adherence barriers reported among participants in similar studies conducted elsewhere. Prior to this study, we did not find studies conducted on barriers to ART adherence among ART non-adherent participants in Cameroon, but studies elsewhere have reported similar barriers such as food insecurity [33, 34], stigma related disclosures [26, 35, 36], low social support and poor relationship between patient and caregivers [37–39], mental illness (especially depression) [40, 41], intrapersonal and cognitive issues (perceived psychological and behavioral issues) [42, 43], preference for traditional medicine and forgetfulness [26, 27], and side effects of ARVs [44]. In contrast, other studies have reported additional barriers to ART adherence such as substance abuse, denial of HIV status, treatment longevity, patients’ socioeconomic status, social isolation and complexity of regiments [38, 45, 46], which were not documented in this study. This study has however reported political instability and insecurity (ghost towns, roadblocks and gunshots) as a major barrier to ART adherence experienced by patients especially in the Northwest and Southwest regions of Cameroon which have not been reported previously. During political instability, there is disruption of health care and displacement of patients–limiting patients’ access to ART. Unplanned prolonged ART interruption caused by ghost towns for instance have negative effects on HIV treatment outcomes including increased morbidity and mortality, as well as development of drug resistant strains of the virus [23]. Besides political instability, the COVID-19 pandemic also had a major impact on ART adherence. The pandemic disrupted HIV care [24, 47] through lockdowns, physical distancing and isolation restrictions measures that were enforced. COVID-19 restrictions also caused fear and anxiety symptoms in patients resulting in low ART adherence [47–49].

Concerning facilitators to ART adherence reported in this study, other studies have documented similar factors facilitating ART adherence such as use of support groups and social networks [20, 22, 50], support from health care providers, use of reminder aids [51, 52], linking pill-taking with daily activities or events [44, 53], and social support [19]. Nevertheless, other studies have also documented additional ART adherence facilitators such as perceived need to meet family responsibilities, perceived positive outcomes, self-motivation, disclosing HIV status to others, responsibility for raising children and experienced treatment benefits [19, 50, 51, 53].

### Limitations and strengths of study

The study included only a cross section of PLWH at the treatment centres who acknowledged that they had not been adherent to treatment. This might not be representative of the population of patients that do not adhere to treatment in the country. Thus, results of the study may not be generalizable to other communities in Cameroon. Again, there is the possibility of response bias as participants may not have revealed the real picture of their past adherence to treatment history. There may equally be recall bias as participants may not have fully remembered their reasons for non-adherence to ART or whether they had been non-adherent to HIV treatment or not. Also, we were looking for reasons for non-adherence which change over time; it is possible that respondents did not offer the full range of their reasons or reported only recent reasons for non-adherence.

Nonetheless, the strengths of the study are that it was conducted in multiple randomly selected settings reflecting the diversity of patients in the country, interviewers were trained to ensure adequate data collection and primary data that ensures that questions pertaining to this study are directly ascertained were collected not just from PLWH but precisely from those who acknowledged they had experienced non-adherence with ART.

## Conclusion

The barriers to ART adherence in Cameroon include patient barriers (forgetfulness, business with other things, unwillingness to swallow drugs daily), medication barriers (side effects of drugs), health service barriers (arrogance of caregivers, occasional drug shortages at treatment centre, poor counseling of patient), stigma related barriers (fear of status disclosure), use of alternative treatment (traditional medicine, prayers and deliverance), resource limitation (limited food, limited finances), environmental/social barriers (limited or no home support), and political instability (disruption of free circulation by ghost towns, roadblocks and gunshots in some regions). ART adherence facilitators include factors such as social support (family and peer support), aligning treatment with patient’s daily routines (align ART with schedule of family members), use of reminders (phone alarm, sound of church bell), health sector/caregiver support (messages to patient, financial support, proper counseling), and patient’s awareness of HIV status/ART knowledge (awareness of HIV positive status, Knowledge of ART benefits). Given these barriers and facilitators, continuous information provision and unflinching support both from patients’ families and caregivers are needed to improve ART adherence among patience. Further studies including many regions and larger samples using both in-depth and focused group discussions as well as quantitative approaches (to determine the scale of interruptions especially in conflict areas using objective measures such as medication refill adherence, viral load, and resistance test results) are required to uncover the burden related to ART nonadherence.

## Supporting information

S1 Checklist PLoS Inclusivity in global research questionnaire. (DOCX)

S2 Checklist COREQ (Consolidated Criteria for Reporting Qualitative Research) 32-item checklist. (DOC)

## List of abbreviations

AIDS: Acquired Immune deficiency syndrome
ART: Antiretroviral Therapy
ARV: Antiretroviral drugs
CD4: Cluster of differentiation 4
COVID-19: Coronavirus disease 2019
CI: Confidence interval
HIV: Human immunodeficiency virus
IQR: Interquartile range
IRB: Institutional Review Board
KM: Kilometers
N: Frequency
LMIC: Low and Middle-Income Countries
PLWH: People living with HIV
SD: Standard deviation
SSA: Sub-Saharan Africa
