# Supplementary material for: Barriers and facilitators to ART adherence among ART non-adherence people living with HIV in Cameroon: A qualitative phenomenological study
Source: PLoS One. 2023 Sep 12;18(9):e0291487. doi: 10.1371/journal.pone.0291487 (PMC10497158; doi:10.1371/journal.pone.0291487)
Supplement: S2 Checklist — (DOC) [file pone.0291487.s002.doc]

**COREQ (Consolidated Criteria for Reporting Qualitative Research)**

**32-item checklist**

## Manuscript: Barriers and facilitators to ART adherence among ART non-adherence people living with HIV in Cameroon: a qualitative phenomenological study

.

**Developed from:** Tong A, Sainsbury P, Craig J. Consolidated criteria for reporting qualitative research (COREQ): a 32-item checklist for interviews and focus groups. *International Journal for Quality in Health Care*. 2007. Volume 19, Number 6: pp. 349 – 357

| **No. Item** | **Guide questions/description** | **Reported on Page #** |
| --- | --- | --- |
| **Domain 1: Research team and reﬂexivity** | | |
| *Personal Characteristics* | | |
| 1. Inter viewer/facilitator | Which author/s conducted the interview or focus group? | Page 7  (AB) |
| 2. Credentials | What were the researcher’s credentials (e.g., PhD, MD)? | (Graduate Student) |
| 3. Occupation | What was their occupation at the time of the study? | (Public Health Student) |
| 4. Gender | Was the researcher male or female? | AB  (male) |
| 5. Experience and training | What experience or training did the researcher have? | Page 7  (Public Health Student/ Interview Training) |
| *Relationship with participants* | | |
| 6. Relationship established | Was a relationship established prior to study commencement? | Page 9  (When obtaining consent) |
| 7. Participant knowledge of the interviewer | What did the participants know about the researcher (e.g., personal goals, reasons for doing the research)? | Participants were informed about researcher and objectives of study during the consenting process |
| 8. Interviewer characteristics | What characteristics were reported about the interviewer/facilitator (e.g., bias, assumptions, reasons and interests in the research topic)? | Page 6  (Trained interviewers with background training in health). |
| **Domain 2: study design** | | |
| *Theoretical framework* | | |
| 9. Methodological orientation and Theory | What methodological orientation was stated to underpin the study (e.g., grounded theory, discourse analysis, ethnography, phenomenology, content analysis)? | Page 5  (Qualitative phenomenology) |
| *Participant selection* | | |
| 10. Sampling | How were participants selected (e.g., purposive, convenience, consecutive, snowball)? | Page 6  (Purposeful) |
| 11. Method of approach | How were participants approached (e.g., face-to-face, telephone, mail, email)? | Page 6, 7  (Face-to-face) |
| 12. Sample size | How many participants were in the study? | Page 6  (Forty-three) |
| 13. Non-participation | How many people refused to participate or dropped out? What were their reasons? | Not applicable  (0) |
| *Setting* | | |
| 14. Setting of data collection | Where was the data collected (e.g., home, clinic, workplace)? | Page 7  (Arranged office in clinic or treatment centre) |
| 15. Presence of non-participants | Was anyone else present besides the participants and researchers? | Page 7  (No) |
| 16. Description of sample | What are the important characteristics of the sample (e.g., demographic data, date)? | Page 9  (participants’ characteristics) |
| *Data collection* | | |
| 17. Interview guide | Were questions, prompts, guides provided by the authors? Was it pilot tested? | Page 7  (Yes) |
| 18. Repeat interviews | Were repeat interviews carried out? If yes, how many? | Not applicable  (No) |
| 19. Audio/visual recording | Did the research use audio or visual recording to collect the data? | Page 7  (Audio recording) |
| 20. Field notes | Were ﬁeld notes made during and/or after the interview or focus group? | Not applicable  (No) |
| 21. Duration | What was the duration of the interviews or focus group? | 15 – 20 minutes |
| 22. Data saturation | Was data saturation discussed? | Page 6  (Recruitment of participants stopped once saturation was attained) |
| 23. Transcripts returned | Were transcripts returned to participants for comment and/or correction? | Not applicable  (No) |
| **Domain 3: analysis and ﬁndings** | | |
| *Data analysis* | | |
| 24. Number of data coders | How many data coders coded the data? | Page 7  (Two) |
| 25. Description of the coding tree | Did authors provide a description of the coding tree? | Page 29-32  (Tables 2 and 3) |
| 26. Derivation of themes | Were themes identiﬁed in advance or derived from the data? | Page 7  (Derived from the data) |
| 27. Software | What software, if applicable, was used to manage the data? | NVIVO 12 |
| 28. Participant checking | Did participants provide feedback on the ﬁndings? | Page 8  (No -two investigators reviewed the coded data to validate it) |
| *Reporting* | | |
| 29. Quotations presented | Were participant quotations presented to illustrate the themes/ﬁndings? Was each quotation identiﬁed (e.g., participant number)? | Pages 10 - 17  (All participants identified when quoted) |
| 30. Data and ﬁndings consistent | Was there consistency between the data presented and the ﬁndings? | Pages 10-17 |
| 31. Clarity of major themes | Were major themes clearly presented in the ﬁndings? | Pages 29 - 32  (Tables 2 and 3) |
| 32. Clarity of minor themes | Is there a description of diverse cases or discussion of minor themes? | Pages 29 - 32  (Tables 2 and 3) |
